# Supplementary material for: FitMultiCell: simulating and parameterizing computational models of multi-scale and multi-cellular processes
Source: Bioinformatics. 2023 Nov 8;39(11):btad674. doi: 10.1093/bioinformatics/btad674 (PMC10666203; doi:10.1093/bioinformatics/btad674)
Supplement: btad674_Supplementary_Data [file btad674_supplementary_data.zip › FitMultiCell_publication_supp_review2.pdf]

Supplementary information:

FitMultiCell: Simulating and parameterizing computational models of multi-scale and multi-cellular processes

Emad Alamoudi [1], Yannik Schälte [1,2,3], Robert Muller [4], Jörn Starruß [4], Nils Bundgaard [5], Frederik Graw [5,6,7], Lutz Brusch [4], and Jan Hasenauer [1,2,3,*]

[1] Life and Medical Sciences Institute, University of Bonn, 53113 Bonn, Germany

[2] Helmholtz Zentrum München - German Research Center for Environmental Health, Institute of Computational Biology, 85764 Neuherberg, Germany.

[3] Technische Universität München, Center for Mathematics, Chair of Mathematical Modeling of Biological Systems, 85748 Garching, Germany.

[4] Center of Information Services and High Performance Computing (ZIH), Technische Universität Dresden, 01062 Dresden, Germany.

[5] BioQuant - Center for Quantitative Biology, Heidelberg University, 69120 Heidelberg, Germany.

[6] Interdisciplinary Center for Scientific Computing, Heidelberg University, 69120 Heidelberg, Germany.

[7] Friedrich-Alexander-University Erlangen-Nürnberg, Department of Medicine 5, 91054 Erlangen, Germany.

*To whom correspondence should be addressed.

Contents

1 Algorithm implementation of ABC-SMC **2**

2 Implementation and Tools **2**

2.1 Technical specifications 2

2.2 FitMultiCell Software 3

2.3 pyABC Software 3

2.4 Morpheus Software 4

3 Specification of model and estimation problem **5**

3.1 (M1) HCV model 5

3.2 (M2) Tumor Growth Model 6
3.2.1 Fully automatic approach for summary statistics weighting 7
3.3 (M3) Liver regeneration model 8

4 Scaling study **9**

1 Algorithm implementation of ABC-SMC

ABC is a likelihood-free inference method particularly applicable to complex stochastic models, for which likelihood evaluation is computationally prohibitive. This is often the case for multi-scale models [1]. ABC only requires the ability to generate simulated data from the model given input parameters. In a nutshell, ABC calculates a distance between simulated and observed data and accepts corresponding parameters if the distance is below an acceptance threshold. ABC samples from an approximation to the Bayesian posterior distribution, thus providing uncertainty-aware parameter estimates [2]. ABC is frequently combined with an SMC approach, which allows for efficient gradual reduction of the acceptance threshold over a series of particle populations, and which is straightforward to parallelize [3, 4].

There exist various extensions to the core ABC routine, e.g. allowing to auto-tune hyperparameters [5], adapt distances [2] or population sizes [6] to the problem structure, select acceptance thresholds [7, 8], or learn low-dimensional summary statistics [9]. Many such approaches are implemented in pyABC (`https://github.com/icb-dcm/pyabc`), see [4, 10] for details. In particular, such semi-automatic self-tuned and robust approaches make the tool accessible also to users without expert knowledge.

The sampler implementation uses the *Redis* package (`https://redis.io`) as a broker between the main process and workers on a distributed high-performance computing (HPC) architecture.

2 Implementation and Tools

2.1 Technical specifications

For our test we used Anaconda 3, Python 3.8, with package versions pyABC 0.10.15, Morpheus 2.2.5, FitMultiCell 0.0.9. Most analyses were performed on the Juelich Supercomputing Center (JSC), Juwels cluster, standard compute nodes, specification of which are 2× Intel Xeon Platinum 8168 CPU, 2× 24 cores, 2.7 GHz 96 (12 × 8) GB DDR4, 2666 MHz. Each node has 48 cores, equaling the number of workers per node used, as all models were single-threaded.

Table S1: Libraries and algorithms used in FitMultiCell

Name	Function	Web page
pyABC	Framework for distributed, likelihood-free inference	https://github.com/ICB-DCM/pyABC
Morpheus	Modelling and simulation environment for the study of multi-scale and multicellular systems	https://morpheus.gitlab.io
Redis	Message broker and in-memory database	https://github.com/antirez/redis

2.2 FitMultiCell Software

The FitMultiCell pipeline simplifies the process of estimating parameters for non-linear stochastic dynamics, especially when it's unfeasible to calculate the likelihood function of experimental data based on a certain set of model parameters. This tool has proven effective in several multicellular stochastic processes, including scenarios such as tissue regeneration and viral propagation. FitMultiCell focuses on the development of an integrated computational platform for data-driven modelling and parameter inference of multicellular systems, with dedicated work packages for improving the scalability and acceleration of algorithms for simulation and parameter estimation. The codes for parameter inference used in FitMultiCell has been developed in-house (pyABC) and for model development and simulation, we use Morpheus (see table S1. The use of these libraries for our purposes is not limited by any licensing restrictions.

2.3 pyABC Software

pyABC is a statistical inference tool that employs an Approximate Bayesian Computing-Sequential Monte Carlo (ABC-SMC) framework [4]. pyABC supports single-machine multi-core execution and multi-machine distributed execution based on a variety of distributed execution engines, such as ad hoc clusters (e.g., the Dask distributed cluster and the IPython parallel cluster), bare grid-submission systems (e.g., SGE and UGE), and Redis based, low latency setups. One of pyABC's distinguishing features is the offering of three parallelization strategies, Static and Dynamic, and look-ahead Scheduling, for the sampling of particles across individual populations. The Dynamic Scheduling approach stands out as an efficient parallelization strategy that can be scaled up to thousands of cores to minimize runtime. The look-ahead tries to optimize resources further by utilising the idle resources at the end of each population. The software is user-friendly, providing non-experts with well-tested default settings and automatic adaptations for elements such as acceptance threshold schedules, transition kernels, and population sizes. For more advanced users, pyABC allows customization and experimentation with ABC-SMC schemes without the need to modify the source code. Additionally, pyABC offers a web interface for visualizing ongoing and finished runs, and an API for querying and post-processing. Written in Python 3, the tool is available under a 3-clause BSD license, and its source code is accessible on GitHub (https://github.com/icb-dcm/pyabc) and can be installed from the Python Package Index (PyPI). A detailed documentation is avail-

able (https://pyabc.readthedocs.io). pyABC has proven its applicability across a broad spectrum of model types, such as ordinary differential equations, Markov jump processes, and agent-based multi-scale models [4]. It has been used successfully in several studies [11, 6?], including the analysis of a multi-scale model describing 10^6 single cells [11], and is being used by both national and international research groups in various projects. pyABC has repeatedly outperformed other tools (including manual tuning), successfully determining parameter values to provide accurate representations of experimental data where other methods have failed.

2.4 Morpheus Software

Morpheus is an open-source software framework that supports user-friendly declarative modeling and offers a graphical user interface (GUI) based on Qt (Fig. S1). Additionally, it provides the MorpheusML domain-specific modeling language, which allows for easy composition and extension of multicellular models. MorpheusML offers a bio-mathematical language where symbolic identifiers in mathematical expressions describe the dynamics and coupling of different model components. It employs the Cellular Potts Model (CPM) formalism to represent the spatial and mechanical aspects of cell interactions and adheres to the software design principle of separating the model from its implementation. This design allows for model sharing, versioning, and archiving. A numerical simulation is constructed by parsing the MorpheusML model definition and automatically scheduling predefined components in the simulator. Numerous models have been published in a model repository (https://morpheus.gitlab.io/model), enabling users to explore and adopt submodels. In addition to its integration with Morpheus, MorpheusML has become a standard for defining models, facilitating interoperability among multiple simulators, parameter estimation pipelines, and model repositories [12].

The GUI features an automatically generated and interactive model graph of the MorpheusML model definition (Fig. S1, lower right). Boxes represent model components, variables and processes. Each box can be clicked and then the corresponding model element, notably parameter, is highlighted in the model editor panel. That model editor has the ability to create the unique XML path string upon right-click directly from the GUI (Fig. S1-I). This XML path then serves as a unique identifier for that specific parameter within the FitMultiCell pipeline and PEtab-MS file.

To speed up the FitMultiCell pipeline, several performance improvements have been implemented, including multicore parallelization of diffusion (for details see `https://morpheus.gitlab.io/post/2023/01/09/morpheus-2.3-release-notes/#reaction-diffusion-system-performance`) and the CPM model formalism (for details see `https://morpheus.gitlab.io/post/2023/01/09/morpheus-2.3-release-notes/#parallel-cpm`). CPM interactions are scaled based on lattice properties [13]. This simplifies the choice of lattice type and resolution to achieve optimal performance for a given model. Moreover, expressions (of position or of any dynamical model variable) are supported everywhere in the model to also account for heterogeneities in parameters (Fig. S1-II).

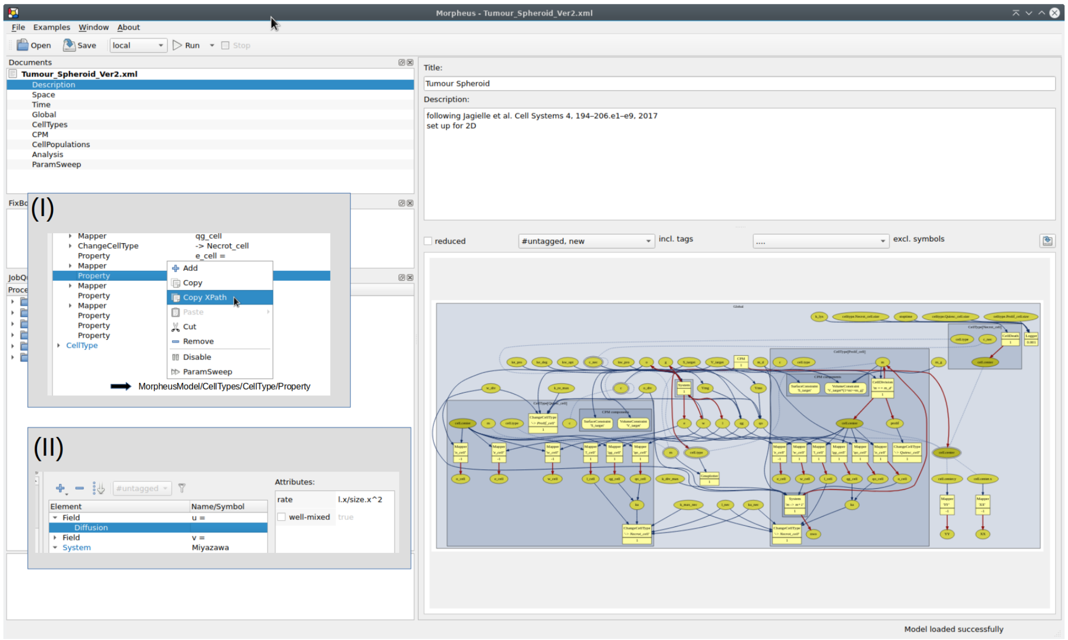

Figure S1: Graphical user interface (main panel) of the Morpheus software. (I): obtaining the XPath for a parameter, (II): spatial expression for diffusion constant.

3 Specification of model and estimation problem

3.1 (M1) HCV model

A model of the spread of the hepatitis C virus among cells was used to test the parallel efficiency of the FitMultiCell pipeline. The model was originally developed by [14] and aims to describe viral spread in a spatially-defined environment by accounting for cell-to-cell (CC) and cell-free infection (CF).

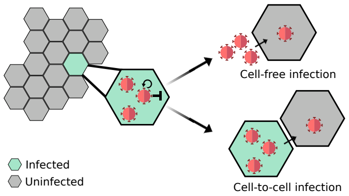

We have re-implemented the model in the FitMultiCell pipeline context. Morpheus was used to generate the MorpheusML model. The new model, similar to the original one, was built using a set of PDEs and ODEs that describes the infection process. A smaller version of the model was created, consisting of 721 cells arranged in a hexagonal grid. This smaller model served primarily for evaluating the performance and scalability of the pipeline. Only two parameters were fitted, as shown in table S2.

The model was fitted to synthetic data and the summary statistics for this model were constructed manually as 1) the number of clusters of infected cells, where a cluster is defined as a connected group of infected cells that initially start small and then increases toward the middle, before reducing again as the entire population becomes infected and forms fewer, larger clusters., 2) the number of cells infected by different modes of transmission (either CC or CF), and finally 3) the number of cells that can still contribute to CC infection. A cell remains CC infectious as long as it stays

Table S2: List of fitted parameters of model M1

Parameter	Description	Prior
$\log_{10}(\text{sf})$	Scaling factor of CF infection	U(-3,-1)
$\log_{10}(\text{sc})$	Scaling factor of CC infection	U(-5,0)

connected to a healthy cell. Given the generic nature and wide application of these three summary statistics, we have made them accessible on FitMultiCell. They can be directly applied during model construction.

A Euclidean distance was used as an objective function. The model was set to run until it reach to acceptance rate that is below 0.01. Each population was set to have a size of 200 accepted parcels.

To ensure the model's reproducibility, a copy of the parameter estimation problem is provided in the Supplementary Material. This includes all its components, such as the distance function, synthetic data, summary statistics, and the observable function, all encapsulated within a PEtab-MS.

3.2 (M2) Tumor Growth Model

The tumor growth model (M2) is our first application to experimental data. The model was developed by [15] and aims to describe the in vitro growth of a tumor spheroid while taking into account its spacial structure.

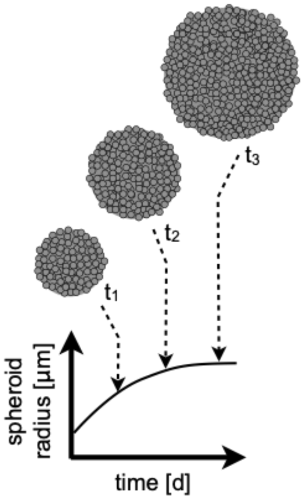

We have re-implemented the model in the FitMultiCell pipeline context. The model was created in Morpheus and encoded as MorpheusML model. In this implementation, we combine a PDE for the description of extracellular matrix density with a CPM for cell migration. In addition, cell division and cell death are implemented using the same mechanisms as in the model by [11]. For a description of the biological processes, we refer to [11]. For a detailed model specification we recommend to open the MorpheusML file in the Morpheus GUI. This will not only provide the model implementation but also information about the implementation of the individual components.

We note that the model is stochastic in nature. In particular while the cell numbers are small – which is the case for early time points – simulated trajectories show a high degree of variability. For large cell numbers a self-averaging effect is observed and the variability is rather low.

Table S3: List of fitted parameters of model M2. All parameters have been transformed to a logarithmic scale with base 10. The posterior mean and 95% credible intervals are based on the results of the last generation of the analysis.

Parameter	Description	Prior	Posterior mean	95% Credible intervals
$\log_{10}(k_{\text{max}}^{\text{div}})$	Division rate	U(-3,-1)	-2.096	(-2.14, -2.04)
$\log_{10}(L_{\text{div}})$	Division depth	U(-5,0)	2.15	(2.10, 2.21)
$\log_{10}(L_{\text{init}})$	Initial spheroid radius	U(1,3)	1.18	(1.17, 1.21)
$\log_{10}(q_{\text{init}})$	Initial quiescent cell fraction	U(0,1.2)	-2.72	(-4.83, -0.27)
$\log_{10}(k_{\text{pro}}^{e})$	ECM production rate	U(-5,0)	-3.38	(-3.45, -3.33)
$\log_{10}(k_{\text{deg}}^{e})$	ECM degradation rate	U(-5,0)	-2.30	(-2.41, -2.23)
$\log_{10}(e_{\text{div}})$	ECM division threshold	U(-5,0)	-2.32	(-2.40, -2.22)

The model we implemented is a two-dimensional cross-section of the tumor spheroid. It possesses a large number of parameters, yet, following the original publication only seven parameters were estimated from the available datasets. These parameters are listed in Table S3. We note that the parameter implementation was conserved in the re-implementation process. The comparison of the parameter estimates which is presented in the main manuscript confirms the consistency.

Following the original manuscript [11], the parameters of the model are estimated from imaging data. Growth curves provide information about the time-dependent size of the spheroid. Histological imaging data were processed to obtain radial profiles, e.g. for the fraction of cells at a certain distance from the spheroid rime which is proliferating. Here, we used the post-processed data provided in [11]. For these data a distance function was formulated based on the weighted ℓ_2 distance, and the weighting was determined using an iterative procedure.

The precise specification of the parameter estimation problem, including the choice of distance function and weighting is provided using PEtab-MS to ensure reproducibility.

3.2.1 Fully automatic approach for summary statistics weighting

In Model (M2), we employ innovative sensitivity weights derived from regression models to measure the informativeness of data on parameters, as detailed in [16]. This approach is already incorporated in the parameter estimation tool pyABC [10]. The regression model can be pre-trained using completed particles, including both accepted and rejected ones, leading to a more efficient implementation by integrating the training phase into the actual ABC-SMC run. The regression model output then helps to assign additional weights to the data based on the informativeness of the underlying parameters. This informativeness is influenced by the sensitivity of the posterior expectation of parameters, or their transformations, given observed data, and how it would change under data perturbations.

Table S4: List of fitted parameters of model M3. All parameters have been transformed to a logarithmic scale with base 10. The posterior mean and 95% credible intervals are based on the results of the last generation of the analysis.

Parameter	Description	Prior	Posterior mean	95% Credible intervals
$\log_{10}$(k1)	Max. flux of SENSOR activation	U(1,3)	2.30	(1.97, 2.54)
$\log_{10}$(k2)	Ratio max. fluxes of SENSOR inact./act.	U(0,2)	0.40	(0.33, 0.45)
$\log_{10}$(k3)	Ratio of YAP synthesis rate/YAP act.	U(-0.76,1.23)	0.23	(-0.10, 0.84)
$\log_{10}$(k4)	Ratio of YAP inactivation rate/YAP act.	U(-1.95,0.04)	-0.54	(-0.71, -0.39)
$\log_{10}$(k5)	YAP activation rate	U(1,4)	2.39	(1.72, 2.91)
$\log_{10}$(k6)	Ratio of inact.YAP to SF binding/unbind.	U(-1.74,0.25)	-0.80	(-1.25, -0.23)
$\log_{10}$(k7)	Inact. YAP unbind. rate from SF	U(1,4)	2.34	(1.62, 3.04)
$\log_{10}$(k8)	YAP export rate from nucleus	U(1,4)	2.44	(1.66, 2.97)
$\log_{10}$(k9)	Ratio of YAP nuclear import/export rate	U(-1.76,0.23)	-0.73	(-0.77, -0.71)
$\log_{10}$(k10)	Ratio of YAP degradation rate/YAP act.	U(-0.95,1.04)	-0.03	(-0.41, 0.041)
$\log_{10}$(k11)	Ratio of inact.YAP degrad. rate/YAP act.	U(0.47,2.47)	1.34	(0.79, 1.88)
$\log_{10}$(k12)	M-M const. of SENSOR activation	U(-4,-2)	-2.82	(-3.53, -2.48)
$\log_{10}$(k13)	M-M const. of SENSOR inactivation	U(-1.6,0.40)	0.07	(-0.27, 0.26)
$\log_{10}$(k14)	Intensity normalization total YAP	U(-1,1)	-0.005	(-0.027, 0.015)

3.3 (M3) Liver regeneration model

We consider a model of YAP regulation by mechanical stimulation through expansion of the bile canaliculi (BC) [17]. Single realisations of the model vary greatly based on parameter values and need runtimes ranging from 3 to 3,000 seconds. This model has 14 unknown parameters and two observables, namely nuclear YAP and total YAP intensities which were quantified from image tiles covering an entire liver lobule with portal and central veins. The details of the 14 parameters are listed in table S4. The yes-associated protein (YAP) is the downstream effector of the Hippo pathway that plays an important role in liver regeneration and developmental size regulation of many organs.

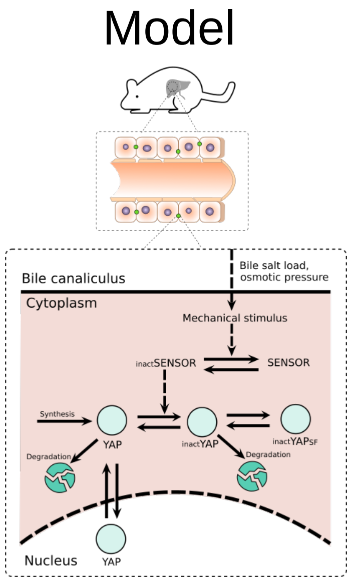

Two sub-models were used to describe the changes in osmotic pressure and the concomitant activation of YAP after partial hepatectomy. The first sub-model is a biophysics-based model to predict the local mechanical stress and apical membrane strain that result from the alteration of osmolyte (bile acid) load in the BC network after partial hepatectomy. It considers the spatial geometry of

the BC within the portal and central vein axis of the lobule. Sub-model 2 is a biochemistry-based model that predicts the cellular response of YAP to the local mechanical stress.

To account for measurement noise [18], additive normally distributed noise was added to the simulations, with a standard deviation of $\sigma = 0.0618$ and $\sigma = 0.0501$ for the two observables NYAP and TYAP, respectively, obtained via average standard errors of the mean from the observed data.

during the fitting process, an Euclidean metric was used as an objective function with a population size set to be 1000 particles. Moreover, the fitting was set to finish after 40 populations. However, most of the runs would be interrupted on around the $16th$ population due to a $24h$ maximum runtime constraint, which already yield robust results. The alpha-quantile of the epsilon threshold was set to 0.30.

Similar to models M1 and M2, all PEtab-MS files for the parameter estimation problem of this model are available on the Supplementary Material.

4 Scaling study

The details of different scenarios used for the scaling study is presented in table S5. The table contains information regarding the number of populations, population sizes, number of nodes/threads, parallel efficiency, wall time in seconds, and speed-ups, total computation time, the total number of simulations performed, and finally, the acceptance rate.

Efficiency and speed-up are indicated relative to runtimes when using 48 cores as a baseline.

Table S5: Overview of scaling behaviour for different scenarios.

Scenario	Number of populations	Size of population	Nodes / cores	Parallel efficiency	Wall time (s)	Speed-up	Total computation time (s)	Total simulations	Ave. acceptance rate
A	3	1000	1 / 48	1	3,623.42	1	173924.16	6878	0.436
A	3	1000	2 / 96	0.99	1,822.43	1.98	174953.28	6735	0.445
A	3	1000	4 / 192	0.88	1,021.42	3.54	196112.64	7290	0.411
A	3	1000	8 / 384	0.69	649.23	5.58	249304.32	8016	0.374
A	3	1000	16 / 768	0.49	453.91	7.98	348602.88	8884	0.337
B	3	10,000	1 / 48	1	33,216	1	1594368	63704	0.470
B	3	10,000	2 / 96	0.99	16,637.43	1.99	1597193.28	64203	0.467
B	3	10,000	4 / 192	0.94	8,778.62	3.78	1685495.04	64199	0.467
B	3	10,000	8 / 384	0.87	4,769.68	6.96	1831557.12	64990	0.461
B	3	10,000	16 / 768	0.75	2,738.99	12.12	2103544.32	65826	0.455

References

[1] J. Hasenauer, N. Jagiella, S. Hross, and F. J. Theis. Data-driven modelling of biological multi-scale processes. *J. Coupled Syst. Multiscale Dyn.*, 3(2):101–121, 9 2015.

[2] Dennis Prangle. Adapting the ABC distance function. *Bayesian Analysis*, 12(1):289–309, 2017.

[3] T. Toni and M. P. H. Stumpf. Simulation-based model selection for dynamical systems in systems and population biology. *Bioinf.*, 26(1):104–110, 10 2010.

[4] Emmanuel Klinger, Dennis Rickert, and Jan Hasenauer. pyABC: distributed, likelihood-free inference. *Bioinf.*, 34(20):3591–3593, 10 2018.

[5] S. Filippi, C. P. Barnes, J. Cornebise, and M. P. Stumpf. On optimality of kernels for approximate Bayesian computation using sequential Monte Carlo. *Stat. Appl. Genet. Mol.*, 12(1):87–107, 2013.

[6] E. Klinger and J. Hasenauer. A scheme for adaptive selection of population sizes in Approximate Bayesian Computation - Sequential Monte Carlo. In J. Feret and H. Koeppl, editors, *Computational Methods in Systems Biology. CMSB 2017*, volume 10545 of *Lecture Notes in Computer Science*. Springer, Cham, 2017.

[7] Christopher C Drovandi and Anthony N Pettitt. Estimation of parameters for macroparasite population evolution using approximate Bayesian computation. *Biometrics*, 67(1):225–233, 2011.

[8] D. Silk, S. Filippi, and M. P. H. Stumpf. Optimizing threshold-schedules for sequential approximate Bayesian computation: Applications to molecular systems. *Stat. Appl. Genet. Mol. Biol.*, 12(5):603–618, Oct. 2013.

[9] Paul Fearnhead and Dennis Prangle. Constructing summary statistics for approximate Bayesian computation: semi-automatic approximate Bayesian computation. *J. R. Stat. Soc. B*, 74(3):419–474, 2012.

[10] Yannik Schälte, Emmanuel Klinger, Emad Alamoudi, and Jan Hasenauer. pyabc: Efficient and robust easy-to-use approximate bayesian computation. *J. Open Source Softw.*, 7(74):4304, 2022.

[11] N. Jagiella, D. Rickert, F. J. Theis, and J. Hasenauer. Parallelization and high-performance computing enables automated statistical inference of multi-scale models. *Cell Syst.*, 4(2):194–206, 02 2017.

[12] Starruß J, et al. *MorpheusML*. FAIRsharing.org, 2023.

[13] Ramiro Magno, Verônica A. Grieneisen, and Athanasius F. M. Marée. The biophysical nature of cells: potential cell behaviours revealed by analytical and computational studies of cell surface mechanics. *BMC Biophysics*, 8(1):8, May 2015.

[14] Peter Kumberger, Karina Durso-Cain, Susan Uprichard, Harel Dahari, and Frederik Graw. Accounting for space—quantification of cell-to-cell transmission kinetics using virus dynamics models. *Viruses*, 10(4):200, Apr 2018.

[15] N. Jagiella. *Parameterization of lattice-based tumor models from data*. Ph.d. thesis, Université Pierre et Marie Curie, Paris, France, 2012.

[16] Yannik Schälte and Jan Hasenauer. Informative and adaptive distances and summary statistics in sequential approximate Bayesian computation. *bioRxiv*, 2022.

[17] Kirstin Meyer, Hernan Morales-Navarrete, Sarah Seifert, Michaela Wilsch-Braeuninger, Uta Dahmen, Elly M Tanaka, Lutz Brusch, Yannis Kalaidzidis, and Marino Zerial. Bile canaliculi remodeling activates yap via the actin cytoskeleton during liver regeneration. *Mol. Syst. Biol.*, 16(2):e8985, 2020.

[18] Yannik Schälte and Jan Hasenauer. Efficient exact inference for dynamical systems with noisy measurements using sequential approximate Bayesian computation. *Bioinf.*, 36(Supplement 1):i551–i559, 7 2020.
